# Needs and preferences of breast cancer survivors regarding outcome-based shared decision-making about personalised post-treatment surveillance

**DOI:** 10.1007/s11764-022-01178-z

**Published:** 2022-02-04

**Authors:** Jet W. Ankersmid, Constance H. C. Drossaert, Yvonne E. A. van Riet, Luc J. A. Strobbe, Sabine Siesling, on behalf of the Santeon VBHC Breast Cancer Group

**Affiliations:** 1 Department of Health Technology and Services Research, Technical Medical Center, University of Twente, Enschede, The Netherlands; 2 Santeon Hospital Group, Utrecht, The Netherlands; 3 Department of Psychology, Health & Technology, University of Twente, Enschede, The Netherlands; 4 Department of Surgery, Catharina Hospital, Eindhoven, The Netherlands; 5 Department of Surgery, Canisius Wilhelmina Hospital, Nijmegen, The Netherlands; 6 Department of Research and Development, Netherlands Comprehensive Cancer Organisation (IKNL), Utrecht, The Netherlands

**Keywords:** Follow-up, Surveillance, Breast cancer, Shared decision-making, Risk information, Personalised care

## Abstract

**Purpose:**

In this study, we explored how patients experience current information provision and decision-making about post-treatment surveillance after breast cancer. Furthermore, we assessed patients’ perspectives regarding less intensive surveillance in case of a low risk of recurrence.

**Methods:**

We conducted semi-structured interviews with 22 women in the post-treatment surveillance trajectory in seven Dutch teaching hospitals.

**Results:**

Although the majority of participants indicated a desire for shared decision-making (SDM) about post-treatment surveillance, participants experienced no SDM. Information provision was often suboptimal and unstructured. Participants were open for using risk information in decision-making, but hesitant towards less intensive surveillance. Perceived advantages of less intensive surveillance were: less distressing moments, leaving the patient role behind, and lower burden. Disadvantages were: fewer moments for reassurance, fear of missing recurrences, and a higher threshold for aftercare for side effects.

**Conclusions:**

SDM about post-treatment surveillance is desirable. Although women are hesitant about less intensive surveillance, they are open to the use of personalised risk assessment for recurrences in decision-making about surveillance.

**Implications for Cancer Survivors:**

To facilitate SDM about post-treatment surveillance, the timing and content of information provision should be improved. Risk information should be provided in an accessible and understandable way. Moreover, fear of cancer recurrence and other personal considerations should be addressed in the process of SDM.

## Introduction

While the incidence of breast cancer is rising in the Netherlands, survival rates have improved due to early detection and improved treatment [1]. These improvements have led to an increase in the prevalence and the number of breast cancer patients receiving follow-up care after curative treatment. Follow-up care can be subdivided into *aftercare* and *post-treatment surveillance*. *Aftercare* primarily focusses on information provision, guidance, identification and dealing with complaints, symptoms, and physical or psychosocial effects of the disease and treatment [2]. The primary aim of *post-treatment surveillance* is early detection of a locoregional recurrence or a second primary tumour [2].

Unlike the highly personalised treatment, post-treatment surveillance is currently one-size-fits-all. The Dutch national guideline recommends an annual mammography and physical examination for at least 5 years after treatment for all curatively treated breast cancer patients (all stages) in a hospital setting [2]. In the Netherlands, surveillance and aftercare are organised by the hospitals. Patients receive invitations for imaging and consultations. Imaging is planned either immediately after the previous imaging or patients receive an invitation by letter or e-mail. Currently, GPs are not actively involved, although there are some initiatives that examine the potential role of primary caregivers in the follow-up.

The effectiveness of the one-size-fits-all approach for surveillance has been a topic of discussion for years. For some women with a low risk for recurrences, it would be sufficient to provide less intensive surveillance than recommended in the Dutch national guideline [3]. Furthermore, more intensive surveillance does not lead to a better health-related quality of life (HRQoL), earlier detection of recurrence, or better survival than less intensive surveillance [4–6]. Therefore, post-treatment surveillance could be individualised based on patient characteristics or disease specifications.

Shared decision-making (SDM) is promoted as the preferred way of medical decision-making, especially for so-called preference-sensitive decisions in situations without a clear medical “best option” [7]. SDM can be defined as ‘an approach where clinicians and patients share the best available evidence when faced with the task of making decisions, and where patients are supported to consider options, to achieve informed preferences’ [8]. The scarce studies on SDM about post-treatment surveillance after breast cancer suggest that SDM is rarely applied [9, 10]. Besides, while information provision is an important step in SDM, little is known about the current information provision about post-treatment surveillance nor about the informational needs that patients have. Brandzel et al. (2017) concluded that patients hardly received information about the aims, benefits, and harms of post-treatment surveillance and that many patients did not feel sufficiently informed to participate in decision-making [9].

SDM would not only be facilitated by general information on surveillance, but also by information on the personal risk for recurrences. Estimating this risk may help to identify patients who might benefit less or more from post-treatment surveillance. The 5-year risk for locoregional recurrences after breast cancer can be calculated with the INFLUENCE-nomogram, a validated prognostic model [11, 12]. However, little is known about how this nomogram is used in clinical practice and how patients feel about using personal risk calculations as part of the SDM process on surveillance.

To our knowledge, no study has evaluated patient perspectives on risk-based post-treatment surveillance and on shared decision-making about this surveillance. Therefore, the aim of this study was to explore breast cancer survivors’ perspectives on SDM about personalised post-treatment surveillance supported with information on the risk for recurrences.

## Materials and methods

### Study design and setting

We conducted semi-structured interviews with women who received post-treatment surveillance after breast cancer in seven Dutch hospitals (Santeon hospital group). The Santeon hospitals are large teaching hospitals located in various regions of the Netherlands with dedicated breast centres, which treat about 11% of all Dutch breast cancer patients. This study was conducted in accordance with local laws and regulations. The Medical Research Ethics Committees United confirmed that the study (reference number W19.134) is not subject to the Medical Research Involving Human Subjects Act (WMO).

### Participants and procedures

The study population for this study consisted of women who received post-treatment surveillance after curative treatment for breast cancer. Patients were excluded if they had a genetic pre-disposition related to breast cancer (due to a different surveillance guideline) and if they were incapable to understand Dutch. We planned to recruit at least 15 patients to achieve data saturation [13]. In the Netherlands, early stage breast cancer patients (M0) are mostly surgically treated and therefore followed by the surgery department (by a surgical oncologist or a nurse practitioner from the surgery department) for surveillance [14]. Therefore, we recruited patients by contacting the surgery departments of the participating hospitals. Patients were recruited through convenience sampling: in each hospital, the first author (JA) and the local surgical oncologist or nurse practitioner selected all the eligible patients who had their consultation on one particular day. During their consultations, the selected patients were approached for participation in the study by their healthcare professional (HCP), who gave a broad description of the aim of the study and the process of data collection. On the same day, patients interested in participating were visited by the researcher (JA), who provided information verbally and in writing. The patients were informed that participation was voluntary and that they could withdraw at any time, without stating any reason [15], and they were given time to ask questions. An interview with each participant was planned for a later date. At the time of the interview, the researcher and the participants signed the informed consent forms (ICF) of the participants. The ICFs of the participants who were scheduled to have an interview by telephone were signed on the day of the recruitment after receiving the information about the study.

Interviews with patients took place between October 2019 and February 2020. The interviews lasted about one hour each and were performed by one researcher (JA, PhD Candidate, MSc. in Psychology), who was trained in conducting interviews. All interviews were audio-recorded with prior permission of participants and were transcribed verbatim. Field notes were taken by JA during the interviews.

#### Interview scheme

The topics for the interviews were derived from literature, and the interview scheme was composed by a team of researchers consisting of two health psychologists, two surgical oncologists, and one epidemiologist. The interviews focused on the following topics: (1) current information provision about surveillance; (2) current decision-making about surveillance; (3) preferences for decision-making about surveillance; (4) current use and perspectives on the use of personalised risk-for-recurrences calculations in decision-making about surveillance; and (5) perspectives on less intensive surveillance in case of a low personal risk. An interview guide was used containing questions about each of these topics. Questions were mainly open-ended and non-directive. Most topics started with an open-ended question, followed by prompting questions to gain more specific information. For example, the following question on information provision, “What do you remember about the discussions you had with your doctor / nurse specialist about post-treatment surveillance?”, was followed by the prompting question “Which information did you receive?”. Moreover, prompting questions were formulated two-directionally, for example, by asking about both the advantages and the disadvantages of less intensive post-treatment surveillance. At the start of each interview, the patient was provided with a written version of the definitions (based on the guideline) of follow-up, aftercare, and post-treatment surveillance to be able to focus on post-treatment surveillance in the interviews.

### Data analysis

Data were analysed using Atlas.ti 9. Transcripts were coded by two independent coders (JA and CD) and analysed using the ‘framework methodology’ [16], which consists of a combination of inductive and deductive approaches. The topics mentioned above formed the basis of the thematic framework. Within each main topic, the coders inductively searched for themes that emerged from the data. They discussed their individual findings several times, and any differences in coding were discussed until consensus was reached.

## Results

### Participants

Of a total of 24 invited patients, 2 declined the invitation to participate. Table 1 shows the baseline characteristics of all the participants. The average age of the 22 participants (all female) at the time of the interview was 59 years. On average, the period since completion of their primary treatment was 3.5 years. Tumour and treatment characteristics of the participants varied.

**Table 1:** Baseline characteristics (*n* = 22)

Demographics	
Age, mean (range), years	59 (29–78)
Tumor characteristics	
Differentiation grade (Bloom–Richardson), *n* (*%*)	
• Grade I • Grade II • Grade III • Missing	6 (27.3%) 13 (59.1%) 1 (4.5%) 2 (9.1%)
Tumor stage (pT stadium, pathological), *n* (*%*)	
• 1 • 2 • pTis • Missing	11 (50%) 7 (31.8%) 2 (9.1%) 2 (9.1%)
Nodal stage (pN stadium, pathological), *n* (*%*)	
• 0 • 1 • 2 • Missing	16 (72.7%) 3 (13.6%) 1 (4.5%) 2 (9.1%)
Multifocality, *n* (*%*)	5 (22.7%)
Hormone receptor (ER/PR) positive, *n* (*%*)	18 (81.8%)
Her2neu receptor positive, *n* (*%*)	2 (9.1%)
Treatment characteristics	
Years since completion primary treatment, mean (range)	3.5 (0.17–8.5)
Type of surgery, *n* (*%*)	
• Lumpectomy • Mastectomy	12 (54.5%) 10 (45.5%)
Neo-adjuvant chemotherapy, *n* (*%*) Adjuvant chemotherapy, *n* (*%*)	2 (9.1%) 7 (31.8%)
Radiotherapy, *n* (*%*)	17 (77.3%)
Hormonal therapy, *n* (*%*) Trastuzumab, *n* (*%*) Immunotherapy, *n* (*%*)	17 (77.3%) 0 (0%) 2 (9.1%)

### Main results

**Fig. 1:**
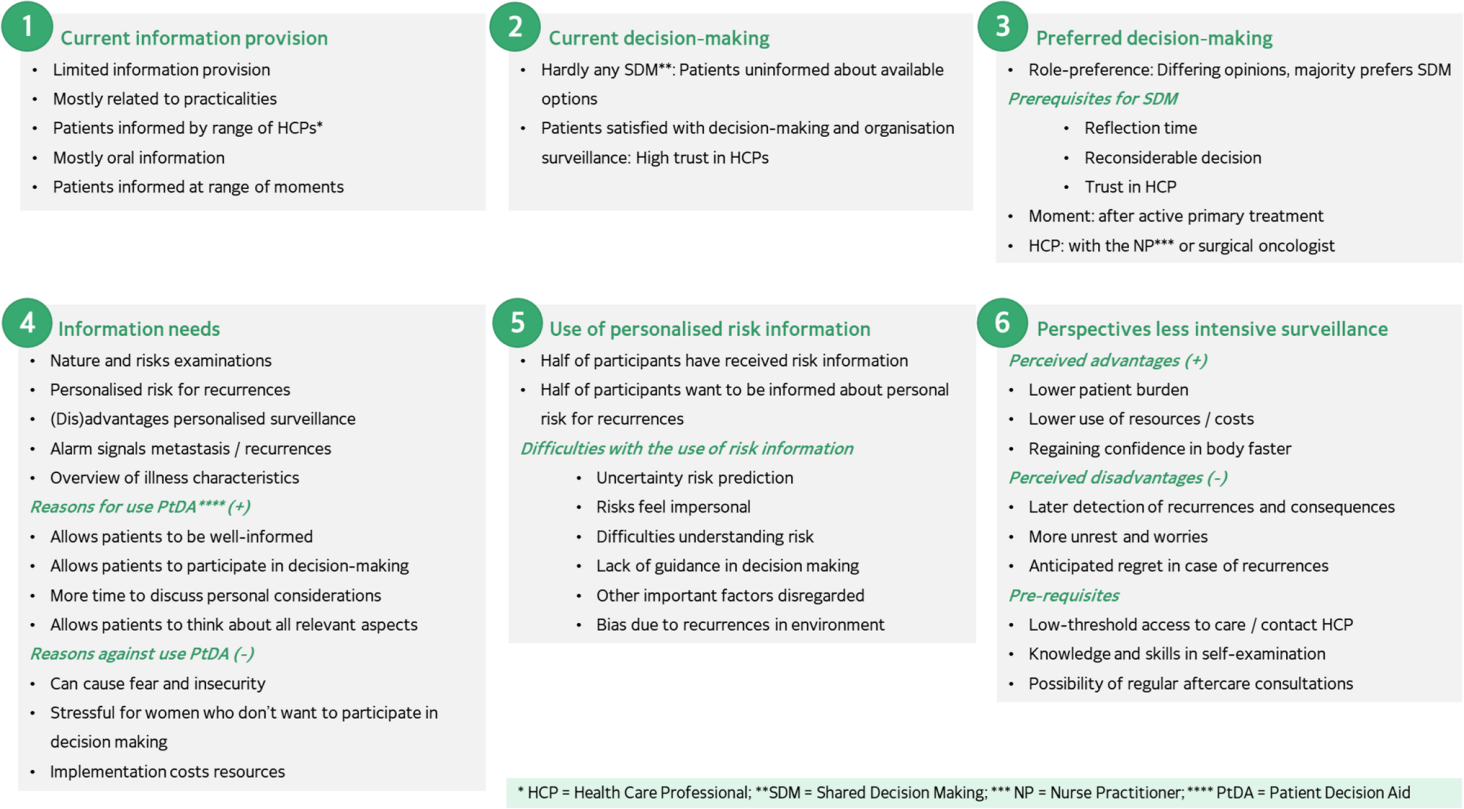
Overview of main themes and subthemes

In total, 20 interviews were held at the seven hospital locations. Two of the interviews took place by telephone for pragmatic reasons. During two of the interviews, a family member of the participant was present (in both cases a daughter), and during one of the interviews, the partner of the participant was present. Data saturation was achieved, because in the last five interviews, no new categories were identified. The main results on each of the topics are summarised in Fig. 1 and discussed below.

#### Current information provision

Information provision about post-treatment surveillance was limited. About half of the interviewed women (*N* = *11*) could not remember receiving any information about post-treatment surveillance: “I don’t remember them mentioning anything about that. This is just how it went” (P1). However, some women (*N* = *7*) indicated that they remembered being informed about surveillance.

For most women (*N* = *13*), the given information was more related to practicalities (when, where, and how) than to aims, benefits, and harms of post-treatment surveillance: “They simply said: After the treatment there is annual surveillance with mammography here in the hospital” (P9). For two women, the provided information was more extensive and also entailed information about the aim of surveillance and the advantages and disadvantages of different types of imaging: “They explained the consequences of an MRI, that it is much more sensitive than a mammography. And that you can get a false positive result and that can also cause tensions” (P6).

Women indicated to have been informed about post-treatment surveillance by different HCPs, ranging from the surgical oncologist to the nurse practitioner of the oncology department, but mainly by the surgical oncologist or by a medical oncologist. Provided information was mostly oral. About half of the interviewed women (*N* = *8*) indicated that written information was provided, but it was not always clear whether this information was specifically focussed on surveillance. The time points at which information provision took place also varied widely and ranged from immediately after the diagnosis to six months after the surgery. Four women indicated that the written information about surveillance was provided too early for their needs (e.g. before the surgery or adjuvant systemic treatment), causing an information overload: “After the diagnosis, I got one leaflet after another. It was too much because I was dealing with the treatments I had to undergo” (P5).

#### Current decision-making

Most of the interviewed women (*N* = *17*) experienced no SDM about post-treatment surveillance: “No, this was simply determined by the hospital” (P3); “I did not feel like I had a choice, but it seemed like a good proposal” (P2). In one case, SDM took place, and the patient indicated that the organisation of post-treatment surveillance was evaluated during each consultation: “Yes, actually we decide every time: if I have different needs, we can adjust the frequency. In choosing between mammography versus MRI versus nothing, I indicated what I thought was important” (P6). In two cases, patients indicated that they were given a choice, but that they left the decision up to the HCP.

Women indicated that they did not know or think about other available options for surveillance: “I also do not know whether there are alternatives in my case” (P9). Even though they did not make a shared decision about post-treatment surveillance, most patients (*N* = *17*) were satisfied about the decision-making process and the current organisation of their surveillance: “No, I didn't feel like I had a choice. This was just protocol, and I was fine with that” (P12). Patients indicated to trust their HCPs and their decisions about what is necessary in terms of their surveillance: “This was determined by the professionals here, and I have confidence in them. If the frequency should be higher, I will hear it from them. And when we can stop, I would love to hear it even more” (P13).

#### Preferred decision-making

Women differed in their opinions on their preferred role in decision-making about surveillance. One woman indicated that she would like to make the decision herself, and another women indicated that she would like to make the decision herself after hearing the opinion of the HCP. The majority of women (*N* = *12*) indicated that they preferred to make the decision together with their HCP (SDM):

“I don't think you can leave it up to the patient alone, because she may have irrational thoughts or wishes that cannot be fulfilled. But the healthcare provider should present the options and ask: What do you prefer?” (P6).

Five patients indicated that they would like to leave the decision to their HCPs after an indication of their preferences: “There will be a certain protocol and the healthcare provider will discuss it with me. And then I can choose to do it differently or a little less often” (P12). Three patients indicated that they would like to leave the decision to their HCPs even without considering the patient’s preference: “I think the doctor can just tell me. I have no knowledge on that anyway” (P13).

As pre-requisites for SDM about surveillance, some women indicated that it is necessary to have reflection time before making the decision (*N* = *3*), that the decision for surveillance should be changeable because feelings or attitudes towards surveillance might change over the years (*N* = *2*), and that patients should trust their HCPs (*N* = *2*).

Patient preferences about the moment when decision-making should take place varied. Most participants (*N* = *19*) agreed that decision-making about surveillance should take place after or at the end of the active primary treatment. The most frequently mentioned reason for this view was that women first need to process their illness, the treatment, and the consequences before they can think about the decision about surveillance: “I would separate treatment and post-treatment surveillance. It would be too much for me. And a decision that I couldn't really cope with at the time” (P4). Even though preferences differed, most women wanted to make a decision about surveillance with their nurse practitioner (NP) (*N* = 7) or surgical oncologist (*N* = *5*). As reasons for preferring decision-making with the NP, women indicated that the NP may have more time than a specialist such as the surgical oncologist that they are accessible and pay attention to the patient as a person. These women indicated that NPs have enough knowledge and skills to guide the SDM process and that they are also under supervision of a specialist:

“I think they [nurses] are more cut out for it. They have the social skills and are easier to approach. It feels less fraught to discuss things with a nurse. I always find a surgeon's time very precious.” (P10).

The women who wanted to decide together with their surgical oncologist mentioned trust and knowledge and skills as the most important reasons: “With the surgical oncologist … I hope she has the knowledge and can recommend the right choice” (P9).

#### Information needs in decision-making

Women indicated several information needs in relation to decision-making about surveillance after breast cancer. Participants indicated that they would like more information about the nature and risks of the examinations (including radiation) (*N* = *8*), about their personalised risk for recurrences and how to deal with fear of cancer recurrence (FCR) (*N* = *5*), about the advantages and disadvantages of personalised surveillance (*N* = *5*), and about the alarm signals for distant metastasis and local recurrences and when to contact an HCP (*N* = *2*). One participant indicated that she would like to have an overview of her illness characteristics, such as the type of cancer and the status of the cancer after treatment.

The majority of women (*N* = 15) indicated that it would be useful to have a patient decision aid (PtDA) for personalised surveillance after breast cancer for several reasons: it allows patients to be well-informed and to actively participate in decision-making, there would be more time to discuss personal considerations during consultations because less time would be spend on information provision (*N* = *5)*, and patients can take the necessary time they to think about the decision and consider aspects that they were not aware of before going through the PtDA (*N* = *3*): “In such a consultation it is possible to discuss your personal considerations more instead of having to get information and having to process the information during the consultation. It helps you prepare” (P6).

As reasons for not using such a PtDA, women mentioned that it might cause fear or insecurity for some patients (*N* = *2)*, that it could be stressful for women that do not want to take part in decision-making (*N* = *1*), and that resources would have to be spent on implementing a PtDA (*N* = *1*).

#### Personal risk calculations in decision-making about post-treatment surveillance

Over half of the participants (*N* = *12*) indicated that they received some form of risk information throughout their treatment for breast cancer. PREDICT [17], a prediction model calculating 5-, 10-, and 15-year survival rates with and without adjuvant systemic treatment, was used for six participants. The outcome of this model was used for discussing the treatment options of chemo- and or anti-hormonal treatment. The Mammaprint [18], a genomic test calculating either a high or low risk for distant metastasis, was requested for three participants. This test was mainly used for discussing the treatment option chemotherapy.

Participants differed in whether they would like to be informed about their risks for recurrences. About half of the participants (*N* = *11*) indicated that they would like to be informed about their personal risk for recurrences because of personal preferences (*N* = *3*), to be able to make decisions (*N* = *2*), to know what to expect in the future (*N* = 2), to be more alert to alarm signals for recurrences in case of high risk (*N* = *1*), or to feel gratitude for recovery (*N* = *1*). Four of the participants indicated that they would not like to be informed about their risk, because the predictions may be uncertain or because knowing the risk may cause fear*.* One participant only wanted to know her risks if it were very high. For the remaining women (*N* = 6), it was unclear whether they would like to be informed, or they were not sure what to think.

Although most of the participants were open for using risk information in decision-making, they did experience some difficulties with the use of risk information in decision-making. These difficulties included finding it hard to deal with the uncertainties of a predicted risk (*N* = *9*), assuming that it can feel impersonal to use risk information (*N* = *2*), fearing that that it can be hard to understand and interpret risk information (*N* = *3*), expecting a lack of attention for other factors that are important for decision-making besides the risk (*N* = *3*), expecting a lack of guidance or advice from the HCP in decision-making (*N* = *3*), or assuming that the risk for recurrences may seem higher in practice than the calculated risk due to recurrences in the social environment (*N* = *2*): “Even if there is only have a half percent chance that something will reoccur, that half percent must represent someone, right? There are people who fall into that half percent” (P4).

#### Perspectives on less intensive post-treatment surveillance

Although some women indicated that they found the use of personalised risks for recurrences in decision-making sensible, many were hesitant about less intensive surveillance in case of a low personal risk.

Perceived advantages of less intensive surveillance were lower patient burden (e.g. fewer stressful moments, fewer negative experiences with hospital visits, pain, time investments and mobility issues) (*N* = *9*), less radiation exposure (*N* = *4*), lower financial costs for patients or care, and thus more resources for patients earlier in the care process (*N* = *9*), and faster recovery of patients’ confidence in their body (*N* = *1*). Perceived disadvantages of less intensive surveillance were later detection of recurrences (*N* = *8*), more unrest and worries (*N* = *8*), and anticipated regret in case of recurrences (*N* = *4*). Some participants described trade-offs between perceived advantages and disadvantages, such as “pain vs. reassurance” and “unrest before and due to annual surveillance vs. the peace of mind that patients gain from reassurance”: “Many women think that a mammography is unpleasant. I don’t think it is fun either, but I will go through the pain to get the reassurance” (P3). Other factors that can be of influence on the decision about less intensive surveillance were prior expectations about the surveillance trajectory (e.g. guidelines or information on the web) (*N* = 4) and expectations of the social environment (e.g. family or friends) (*N* = *2*). Another influencing factor was the attitude of the HCP towards personalised surveillance: “I think it is up to the doctor’s powers of persuasion. How he presents it” (P7).

As prerequisites for less intensive surveillance, participants mentioned low-threshold access to care or contact with an HCP in case of complaints (*N* = *4*), knowledge and skills to perform self-examinations (*N* = *2*), and aftercare consultations at regular intervals (*N* = *1*): “I would like to be sure that care is accessible whenever I have doubts or suspicions or if I am simply worried” (P2).

## Discussion

To our knowledge, this is the first study to examine breast cancer survivors’ perspectives on SDM about personalised post-treatment surveillance supported by information on the risk for recurrences. Our findings suggest that SDM currently does not take place, but that it is deemed desirable by the patients interviewed. Participants were satisfied with their care, but they also indicated that they recognise the preference-sensitive nature of the decision about surveillance as described by de Ligt et al. (2019). Participants saw that surveillance can come at certain “costs” that can be weighed against the benefits, and they also described certain trade-offs.

Although some women indicated that they found it sensible to match the surveillance schedule to personalised risk calculations and that they saw benefits of less intensive surveillance, many were still hesitant regarding less intensive surveillance due to factors such as possible later detection of recurrences, more unrest and worries, and anticipated regret in case of recurrences. Many women indicated FCR as a strong influence on their preference for intensive surveillance. FCR should therefore be addressed in decision-making about surveillance and in general, because research shows that women want to discuss FCR with their healthcare provider [19]. One way to address FCR is to discuss patient-reported outcomes (PROs) regarding FCR. PROs can be discussed at an aggregated or individual level to clarify values and to discuss care needs in the process of SDM [20].

Current information provision about surveillance is often limited to practical information. For SDM, more information is necessary, for example, about the aim of surveillance, the options, and about the advantages and disadvantages of the options. These findings are consistent with findings by Brandzel et al., who found that women did not feel they received sufficient information to participate in decision-making about breast imaging after their treatment [9], and with findings by Shea-Budgell et al. (2014), who found that breast cancer survivors have unmet information needs regarding follow-up [21]. However, these studies did not identify specific topics on which patients should be informed. In our study, participants indicated that they would like to receive information about topics such as self-examination, alarm signals for recurrences, and when to contact whom in case of complaints or worries. In line with Shea-Budgell et al., we advise to develop more comprehensive information materials. Most participants indicated that a PtDA to support decision-making would be useful to support information provision and SDM.

Participants were open to the use of risk information in SDM about surveillance. However, they did not all want to know their risk for recurrences. These findings are similar to the findings of Rainey et al. (2019), who studied healthy British, Dutch, and Swedish women’s perceptions regarding risk‐based breast cancer screening and prevention. Their participants also had mixed feelings about less intensive screening in case of a low risk, but they did want to know their risk for recurrences [22]. The alignment of these findings should be interpreted with caution, since Rainey et al. assessed perspectives of women without prior cancer. In line with Rainey et al., we emphasize the importance of evidence-based strategies and voluntary participation in personalised screening.

### Strengths and limitations

This study has several limitations. Firstly, we interviewed patients that had already received standard follow-up care for some time (average: 3.5 years, range: 2 months to 8 years) so that we could reflect upon the current decision-making process regarding post-treatment surveillance. However, it may have been harder for these patients to imagine the ideal decision-making process for other patients and to consider factors that could play a role in this decision. Secondly, although the number of participants is comparable to that of other qualitative studies, and although saturation was achieved, the number was too small to make useful comparisons between subgroups (e.g. on disease stage, time since completion of primary treatment, age, educational level). These and possibly other variables can influence a patient’s attitude towards SDM and follow-up. Furthermore, the small number of participants led to considerable variation in answers. Quantitative research is needed to achieve more insight into the percentages of women that have certain opinions about the mentioned topics and into the factors that may explain any variation. A large-scale survey could be suitable for this purpose. Thirdly, our study took place in teaching hospitals (non-academic hospitals that educate residents). Surveillance in academic, general, and teaching hospitals may differ slightly, but most hospitals follow the Dutch national guideline. Fourthly, the stage distribution of the patients included in our study seems to be somewhat favourable compared to the general early stage breast cancer (M0) population. However, this makes the results of this study of even more interest because the included patients are part of the patient group for which less intensive surveillance, and therefore, SDM about surveillance is particularly suitable. Finally, results may not be completely generalisable to other countries because surveillance may be organised differently due to cultural or budgetary incentives. However, other countries are also looking to optimize aftercare and surveillance, and the results of this study can contribute to this process [23–25]. Strengths of this study are the fact that data saturation was achieved and the heterogenous group of participants in terms of age, region in the Netherlands, and time since diagnosis and treatment.

### Implications for cancer survivors and care providers

Breast cancer survivors desire SDM about post-treatment surveillance. To facilitate SDM, the timing and content of information provision should be improved. The findings of this study can inform the development of information materials or a PtDA or both, as well as the further design of an SDM process. Risk information should be provided in an accessible and understandable way. Moreover, fear of cancer recurrence and other personal considerations should be addressed in the process of SDM.

## Conclusion

SDM about post-treatment surveillance is desirable. To facilitate SDM, the timing and the content of information provision have to be improved, especially on the aim of surveillance, the options for surveillance and their advantages and disadvantages, on the risks for recurrences, and on self-monitoring and alarm signals for recurrences. Although women are hesitant about less intensive surveillance, they are open to the use of personalised risk assessment for recurrences in decision-making about surveillance. Yet, risk information should be provided in an accessible and understandable way, and fear of cancer recurrence and other personal considerations should be addressed in shared decision-making about post-treatment surveillance.

## Data Availability

The data that support the findings of this study are available from the corresponding author upon reasonable request.

## Appendix A

The Santeon VBHC Breast Cancer Group (collaborators) are C.F. van Uden – Kraan, Santeon Hospital Group, Utrecht, the Netherlands; Y.E.A. van Riet and J.M. Bode-Meulepas, Catharina Hospital, Eindhoven, the Netherlands; L.J.A. Strobbe, Canisius Wilhelmina Hospital, Nijmegen, the Netherlands; A.E. Dassen, Medisch Spectrum Twente, Enschede, the Netherlands; A.F.T. Olieman, Martini Hospital, Groningen, the Netherlands; H.H.G. Witjes, OLVG, Amsterdam, the Netherlands; R. Koelemij, St. Antonius Hospital, Utrecht, the Netherlands; C.M.E. Contant, Maasstad Hospital, Rotterdam, the Netherlands.
